# Supplementary material for: Identification of Freezing-Responsive microRNAs and Their Targets in Chinese Jujube by Small RNA and Degradome Sequencing
Source: Int J Mol Sci. 2026 Sep 4;27(17):7912. doi: 10.3390/ijms27177912 (PMC13566890; doi:10.3390/ijms27177912)
Supplement: Supplementary file 1 [file ijms-27-07912-s001.zip › Supplementary Material S2/novel_miR_118.pdf]

Provisional ID	:	novel_miR_118
Score total	:	0.8
Score for star read(s)	:	-1.3
Score for read counts	:	0
Score for mfe	:	0.5
Score for randfold	:	1.6
Score for cons. seed	:	
Total read count	:	821
Mature read count	:	809
Loop read count	:	12
Star read count	:	0

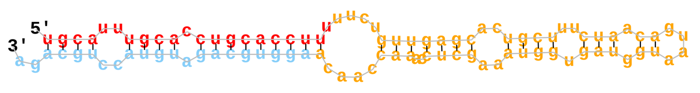

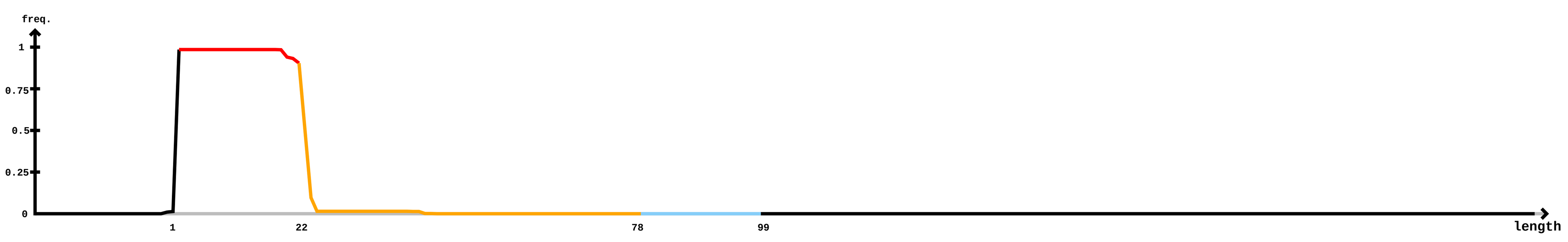

Mature **Star**

```
5'- agccauagugguuucauuuuucugcauuugcaccugcaccuuuuucuguugagcacugcuuucuaacaguaaugguagugguaaagcuccaaaccaacaaggugcagauguaccugcagacaaaugccaccuugguuuccuuuuuucucuuuucucuuccucugcuaccauggccaaucuacgccuuuuuuauguucuauauauccuuucuaguuaacauagauauuaguuuuauuuuaguuggaaguuu -3'   exp
    (((((..((((...(((((.((((((..((((.(((((((((.....(((((((..((((..(((.((....)).))).)))).)))).)))....))))))))).)))).))))))).)))))))))..)))))................(((((...((((....(((........)))...(((((((((((..........)))..))))))))............)))).)))))...   reads   mm   sample
    ....................ucugcauuugcaccugcac...................................................................................................................................................................................................................   1   0   LT-1
    ....................ucugcauuugcaccugcaccu.................................................................................................................................................................................................................   1   0   JCK-3
    ....................ucugcauuugcaccugcaccu.................................................................................................................................................................................................................   3   0   LCK-3
    ....................ucugcauuugcaccugcaccu.................................................................................................................................................................................................................   2   0   LT-1
    ....................ucugcauuugcaccugcacUuu................................................................................................................................................................................................................   1   1   LT-1
    .....................cugcauuugcaccugcaccuuu...............................................................................................................................................................................................................   2   0   JCK-2
    .....................cugcauuugcaccugcaccuuu...............................................................................................................................................................................................................   1   0   JCK-3
    ......................ugcauuugcaccugcacc..................................................................................................................................................................................................................   3   0   JCK-1
    ......................ugcauuugcaccugcacU..................................................................................................................................................................................................................   1   1   LT-3
    ......................ugcauuugcaccugcacc..................................................................................................................................................................................................................   3   0   JCK-2
    ......................ugcauuugcaccugcacc..................................................................................................................................................................................................................   5   0   LT-3
    ......................ugcauuugcaccugcacc..................................................................................................................................................................................................................   13   0   LT-2
    ......................ugcauuugcaccugcacc..................................................................................................................................................................................................................   11   0   LT-1
    ......................ugcauuugcaccugcaccu.................................................................................................................................................................................................................   1   0   LT-1
    ......................ugcauuugcaccugcaccuu................................................................................................................................................................................................................   1   0   JCK-3
    ......................ugcauuugcaccugcaccuA................................................................................................................................................................................................................   1   1   LT-1
    ......................ugcauuugcaccugcaccuu................................................................................................................................................................................................................   4   0   LT-2
    ......................ugcauuugcaccugcaccuu................................................................................................................................................................................................................   2   0   LT-3
    ......................ugcauuugcaccugcaccuu................................................................................................................................................................................................................   1   0   JCK-2
    ......................ugcauuugcaccugcaccuu................................................................................................................................................................................................................   13   0   LT-1
    ......................ugUauuugcaccugcaccuuu...............................................................................................................................................................................................................   2   1   JCK-1
    ......................ugcauuugcaccugcaccuuu...............................................................................................................................................................................................................   138   0   JCK-2
    ......................ugcauuugcCccugcaccuuu...............................................................................................................................................................................................................   1   1   LT-3
    ......................ugcauuugcaccugcaccuuu...............................................................................................................................................................................................................   8   0   JT-2
    ......................ugcauuugcaccugcaccAuu...............................................................................................................................................................................................................   3   1   LT-2
    ......................ugcauuugcaccugcaccuuu...............................................................................................................................................................................................................   71   0   JCK-1
    ......................ugcauuugcaccugcacAuuu...............................................................................................................................................................................................................   1   1   LT-2
    ......................ugcauuugcaccugcaccuuu...............................................................................................................................................................................................................   18   0   JT-3
    ......................ugcauuugcaccugcaccuuu...............................................................................................................................................................................................................   99   0   LT-3
    ......................ugcauuugcaccugcaccuuu...............................................................................................................................................................................................................   62   0   LT-2
    ......................ugcauuugcaccugUaccuuu...............................................................................................................................................................................................................   2   1   JCK-2
    ......................ugcauuugcaccugcaccuuu...............................................................................................................................................................................................................   113   0   JCK-3
    ......................ugcCuuugcaccugcaccuuu...............................................................................................................................................................................................................   2   1   LT-2
    ......................ugcauuugUaccugcaccuuu...............................................................................................................................................................................................................   1   1   JCK-1
```

```
                      Mature                                                                       Star
agccauagugguuucauuuuucugcauuugcaccugcaccuuuuucuguugagcacugcuuucuaacaguaaugguagugguaaagcuccaaaccaacaaggugcagauguaccugcagacaaaugccaccuugguuuccuuuuuucucuuuucucuuccucugcuaccauggccaaucuacgccuuuuuuauguucuauauauccuuucuaguuaacauagauauuaguuuuauuuuaguuggaaguuu
......................ugcCuuugcaccugcaccuuu...............................................................................................................................................................................................   1    1    JCK-2
......................ugcauuugcaccugcaccuuu...............................................................................................................................................................................................   30   0    LCK-3
......................ugcauuugcaccugcaccuuu...............................................................................................................................................................................................   118  0    LT-1
......................ugcauuugcaccugcaccuuuu..............................................................................................................................................................................................   1    0    LT-2
......................ugcauuugcaccugcaccuuuuA.............................................................................................................................................................................................   29   1    LCK-2
......................ugcauuugcaccugcaccuuuuC.............................................................................................................................................................................................   1    1    LT-2
......................ugcauuugcaccugcaccuuuuu.............................................................................................................................................................................................   4    0    LT-2
......................ugcauuugcaccugcaccuuuuA.............................................................................................................................................................................................   33   1    LCK-1
......................ugcauuugcaccugcaccuuGuu.............................................................................................................................................................................................   1    1    LT-3
...........................................uucuguugagcacugcuG.............................................................................................................................................................................   1    1    LT-3
...........................................uucuguugagcacugcuuuc...........................................................................................................................................................................   10   0    LCK-3
.............................................cuguugagcacugcuuucua.........................................................................................................................................................................   1    0    LT-1
```
